# *Toxocara* Seroprevalence and Risk Factor Analysis in Four Communities of the Wiwa, an Indigenous Tribe in Colombia

**DOI:** 10.3390/microorganisms9081768

**Published:** 2021-08-19

**Authors:** Patrick Waindok, Simone Kann, Andrés Aristizabal, Juan Carlos Dib, Christina Strube

**Affiliations:** 1 Institute for Parasitology, Centre for Infection Medicine, University of Veterinary Medicine Hannover, 30559 Hanover, Germany; patrick.waindok@tiho-hannover.de; 2 Medical Mission Institute, 97074 Würzburg, Germany; simone.kann@medmissio.de; 3 Tropical Health Foundation, Santa Marta 470003, Colombia; ajaristizabal@fspt.co (A.A.); jdibdiaz@uninorte.edu.co (J.C.D.); 4 Departement of Medicine, Universidad del Norte, Barranquilla 081001, Colombia

**Keywords:** toxocarosis, toxocariasias, soil-transmitted helminths, zoonosis, roundworms, IgG, ELISA

## Abstract

The life of the indigenous Wiwa tribe in northeast Colombia is characterized by lacking access to clean drinking water and sanitary installations. Furthermore, free-roaming domestic animals and use of yucca and/or manioc as a primary food source favor the transmission of soil-transmitted helminths, e.g., *Toxocara* *canis* and *Toxocara* *cati*, the roundworms of dogs and cats. Infection may result in the clinical picture of toxocarosis, one of the most common zoonotic helminthoses worldwide. To estimate the *Toxocara* seroprevalence in four different villages of the Wiwa community, serum samples from 483 inhabitants were analyzed for anti-*Toxocara*-antibodies. Overall, 79.3% (383/483) of analyzed samples were seropositive. Statistically significant differences were observed between the four villages, as well as age groups (adults > adolescents > children), while sex had no effect. The high seropositivity rate demonstrates the risk of zoonotic roundworm infections and potential clinical disease in vulnerable indigenous inhabitants.

## 1. Introduction

The Sierra Nevada de Santa Marta in the northeast of Colombia presents a great variety of climates and tropical ecosystems within a relatively small area. The ethnohistorical habitation of the region dates back at least to the 1st century AD with the pre-Columbian culture of the Tairona, of which the Kankuamo, Kogi, and Arhuacos, as well as the Wiwa, are the believed direct descendants [1]. The indigenous tribe of the Wiwa have suffered constant violations of their human rights and have historically been victims of, for example the Spanish colonial system, or illegal armed actors for the illicit cultivation of coca, poppy, and marijuana, but also of legal investors with great interest in the indigenous territory [2]. To avoid increasing confrontations, parts of the population moved to higher areas of the Sierra Nevada de Santa Marta, where the Wiwas live a secluded life, with a deep-rooted cultural and spiritual identity with the nature and wildlife surrounding them. Despite growing modernization and tourism in the territory, the Wiwa community strive to keep their ancient culture alive [2]. They live in small communities comprising 200–700 inhabitants per village, in which domestic animals have unrestricted access, even to the people’s living areas in simple clay huts. This, together with the use of natural resources such as yucca and/or manioc and home-grown vegetables as primary food sources, favors the transmission of soil transmitted helminths (STHs) such as *Toxocara canis* and *Toxocara cati*, the zoonotic roundworms of dogs and cats. Furthermore, a lack of access to clean water and sanitary installations affects environmental hygiene as well as personal hygienic standards, providing ideal transmission possibilities for STHs [3,4]. Consequently, high infection rates with *Ancylostoma* spp., *Necator americanus*, *Strongyloides stercoralis*, *Ascaris lumbricoides*, *Trichuris trichiura*, and *Hymenolepis nana* were detected in members of the Wiwa community [5].

Additionally, environmental conditions, such as annual mean temperatures of 25–30 °C, high humidity, and soil moisture, are favourable for the development and tenacity of a broad range of (zoonotic) helminth eggs, such as *Toxocara* spp. [6,7,8]. Consequently, a particularly high seroprevalence of anti-*Toxocara* antibodies in humans is reported for some tropical and subtropical regions or countries [9]. Toxocarosis is one of the most common zoonotic helminthoses worldwide [10], and human infections are primarily acquired after accidental ingestion of embryonated eggs containing infective *Toxocara* spp. third-stage larvae (L3) in contaminated food, water, and soil, or via consumption of L3 in undercooked meat of paratenic hosts such as chicken [11]. By excreting eggs into the human environment, domestic dogs and cats play a pivotal role in the epidemiology of human toxocarosis [12].

Infections may remain asymptomatic; however, the migration and persistence of larvae in the tissues can also result in clinical disease, ranging from mild to severe, and rarely fatal organ injuries [13]. Overall, the disease is classified into four different syndromes. The visceral larva migrans (VLM) is a systemic disease caused by larval migration through major organs, while the covert toxocarosis represents a less severe form of VLM [14]. Larval migration into the eye or the optic nerve manifests in the ocular larva migrans (OLM), while neurotoxocarosis (NT) results from invasion of *Toxocara* larvae into the brain [15,16]. OLM and NT in particular may have severe consequences such as impaired vision and even blindness, or neurological deficits and neuropsychological disorders, exhibiting a lifelong burden, particularly in children [17,18]. The mostly rather unspecific symptoms, along with a lack of knowledge of the disease, are probably the main reasons for underestimating and underdiagnosing toxocarosis, which has been targeted by the US American Centers for Disease Control and Prevention (CDC) as one of the five neglected parasitic infections with priority for public health action [19]. Thus far, to our knowledge, nothing is known about the inhabitants’ health status with regard to *Toxocara* spp. As clinically apparent toxocarosis and its consequences affect the life of the individuals, the presented study aimed to estimate the seroprevalence in the indigenous tribe of the Wiwa, and to analyse potentially related risk factors. The results are necessary to gain knowledge about the risk of exposure, and to raise awareness for this neglected disease in indigenous communities.

## 2. Materials and Methods

### 2.1. Ethical Statement

Sample material was obtained in accordance with the international guidelines of the Declaration of Helsinki, approved by the local ethics committee of Valledupar, Cesar, Colombia (Acta no. 0022013). Written informed consent was obtained from each participant or the parent/legal guardian of a child prior to participation.

### 2.2. Sampling

In July and November 2014, blood samples of 483 indigenous volunteers from the Wiwa villages of Tezhumake (*n* = 173), Ashintukwa (*n* = 106), Cherua (*n* = 94), and Siminke (*n* = 110) were collected. Participants were categorized by their age (children: <6 years, *n* = 75; adolescents: 6–17 years, *n* = 217; adults: >17 years, *n* = 191) and sex (males: *n* = 247; females: *n* = 232). Furthermore, the villages were categorized regarding altitude, being categorized as below 1000 m above sea level (MASL) (Tezhumake: 700 MASL; Ashintukwa: 750 MASL; *n* = 279 inhabitants) and above 1000 MASL (Cherua: 1300 MASL; Siminke: 1100 MASL; *n* = 204 inhabitants). Collected blood samples were cooled in a specific cooling box at 4 °C and transported immediately to the laboratory for serum extraction. Sera were stored thereafter at −20 °C and transferred to Germany with a permanent cooling chain. After arrival, sera were stored at −20 °C until further analysis.

### 2.3. ELISA

To determine anti-*Toxocara* antibodies in the serum samples, the *Toxocara canis* IgG ELISA (product code: DE0450; Demeditec Diagnostics GmbH, Kiel, Germany), based on *Toxocara* excretory-secretory products (TES) as diagnostic antigen, was conducted in accordance with the manufacturer’s instructions. The described diagnostic specificity and sensitivity is given as 98.63% (95%-CI (96.52–99.62%)) and 96.92% (95%-CI (89.32–99.63%)). All runs included a positive and negative control, as well as a calibrator for semi-quantitative analysis. Briefly, all samples were diluted 1:101 with supplied IgG sample diluent, and a volume of 100 µl standards/controls as well as diluted sera were incubated as duplicates for 1 h at 37 °C. After incubation and washing steps consisting of three times 300 µl washing buffer, 100 µl horseradish peroxidase-labelled conjugate was added and again incubated for 30 min at 37 °C. After washing as described, colorimetric detection was initiated by applying 100 µl tetramethylbenzidine substrate solution for 30 min at room temperature and stopped with 100 µl stop solution. The optical density (OD) was measured using a Biowave 340 photometer (BioTek Instruments, Inc., Winooski, VT, USA) at a wavelength of 450 nm as well as a reference wavelength of 620 nm to exclude background signals. Result in units (U) were calculated, and samples were tested negative if U was <9, equivocal if U was ≥9 to <11, or positive if U was ≥11.

### 2.4. Statistical Analyses

Statistical analyses were conducted in R version 4.0.2 [20]. To determine *Toxocara* seroprevalence differences in the Wiwa, a general linear model (GLM) with binomial error structure and logit link function was conducted. Village, age group, and sex were included as fixed factors, and multiple comparisons between these factors were carried out using Tukey HSD contrasts with single-step *p*-value adjustment (package “multcomp” [21]). Final models were compared to null models containing only an intercept term (GLM) in a likelihood ratio test. As comparison of meters above sea level (MASL) could not be fitted in the multiple comparison analyses, villages were categorized according to their location in low altitude below 1000 MASL and high altitude above 1000 MASL, and comparison was repeated with MASL instead of village as a factor.

## 3. Results

### 3.1. Toxocara Seroprevalence in the Wiwa

Anti-*Toxocara* antibodies were detected in 79.3% of the 483 analyzed samples, and in 3.3%, the results were equivocal. In case of equivocal results, the manufacturer recommends repetition with freshly obtained serum after 2 to 4 weeks. As repetition of equivocal results was not possible, these samples were judged as negative to fit in the GLM. 

The highest seroprevalence was detected in the village of Siminke (90.0%), the lowest in Tezhumake (70.5%, Table 1). Subgroup analyses showed a seroprevalence of 66.7% and 76.0% in children and adolescents compared to 88.0% in adults. Regarding sex, 75.7% of participating males and 83.2% of females tested positive. Furthermore, inhabitants of villages below 1000 MASL (Tezhumake and Ashintukwa) exhibited a lower seroprevalence than those living above 1000 MASL (Siminke and Cherua). 

### 3.2. General Linear Modelling

*Toxocara* seroprevalences differed significantly between the communities. Overall, the seroprevalence in villages below 1000 MASL (Tezhumake and Ashintukwa) was statistically significantly lower compared to villages above 1000 MASL. Statistically significant differences were also determined between the age classes: the seroprevalence in adults was significantly higher than in adolescents and children, while sex had no effect on seropositivity. Detailed results are presented in Table 2. 

## 4. Discussion

The worldwide distribution of *Toxocara* spp., their high reproduction rate, and the high tenacity of the eggs result globally in soil contamination of human environments [22,23], making toxocarosis one of the most common zoonotic helminthoses worldwide [10]. As tropical and subtropical conditions with year-round temperatures of >18 °C, high precipitation, and consequently high humidity favor the survival of *Toxocara* eggs, soil contamination rates are elevated at lower latitudes (0–20°) compared to higher ones (41–60°) [22]. Consequently, southeast Asia and South America are salient areas of *Toxocara* prevalence with, estimated on the population size, high burdens of human toxocarosis [24]. Colombia is, with 47.5%, regarded as the country with the highest human seroprevalence in Latin America, even though only one municipality of Bogota was considered in the conducted meta-analysis [24]. However, the result is strengthened by Mendoza Meza [25], as 42.1% of examined schoolchildren in the city of Santa Marta at the Caribbean coast of north Colombia exhibited anti-*Toxocara* antibodies. Notably, the seroprevalence of 79.3% in the northeast Colombian indigenous Wiwa tribe investigated in the present study is remarkably higher than previously described for Colombia.

Besides tropical climate conditions, various further potential risk factors have been associated with human *Toxocara* infections, such as living in rural areas, having contact with dogs, cats, or soil, and the drinking of untreated water [24]. As the Wiwas live retracted in the mid-highlands of the Sierra Nevada de Santa Marta, their life is defined by a lack of access to clean drinking water (rivers and unprotected wells are used as a water source) or sanitary installations [1]. Their lifestyle, together with different cultural aspects such as top dressing, affects the environmental hygiene and hygienic standards of the residents [3,4]. Furthermore, companion as well as stray dogs and cats have unrestricted access to the villages and living areas, and represent a continuous source of environmental egg contamination [26]. Such high soil contamination may lead to contamination of vegetables grown close to the ground. Accordingly, *Toxocara* egg contamination of different vegetables such as lettuce, carrots, potatoes, zucchini, spinach, cucumber, or cress have been described [27,28,29]. The Wiwa use yucca and manioc in combination with home-grown vegetables as a primary food source, and inadequately washed foods or foods washed with contaminated water can be a source for *Toxocara* infections.

The seroprevalence in the Wiwa population varied between the different villages. The seroprevalences of 88% and 90% in inhabitants of Cherua and Siminke were significantly higher compared to those of Tezhumake and Ashintukwa (70% and 75%). Although all communities have difficult living conditions, regional climatic conditions differ between the four villages. The daily average temperature in all four communities is above 18 °C, but Tezhumake and Ashintukwa are located at 700 and 750 MASL, and the climate at this altitude is characterized by a prolonged dry season of up to six months a year. In contrast, Cherua and Siminke are located at 1300 and 1100 MASL, where higher precipitation and higher average humidity and a subtropical or premontane humid forest result in an increased soil moisture. This favors the development and survival of embryonated *Toxocara* eggs, which may survive for up to four years in warm and humid environments [30,31,32]. Thus, climatic and associated abiotic factors may explain the observed seroprevalence differences between the villages.

Furthermore, *Toxocara* seropositivity in the Wiwas was associated with increasing age. However, age as a risk factor for human seropositivity remains debatable. Although children are regarded as being at high infection risk due to a less developed hygienic awareness compared to adults [9,33], elevated levels of anti-*Toxocara* antibodies in elderly people may result from a cumulative effect of *Toxocara* exposure during their lifespan. The constant rechallenge of the immune response due to less developed hygienic standards, leading to frequent and/or high *Toxocara* exposure of all inhabitants regardless of age, may result in the accumulation of detectable antibodies. On average, IgG antibodies against *Toxocara* can be detected for 2.7 years with TES-ELISA [34] as used in the present study, and in many patients requiring chemotherapeutic treatment, IgG persistence lasts over four years [34,35,36]. Nevertheless, about 67% seroprevalence in the Wiwa children is alarming, as toxocarosis can exhibit detrimental long-term consequences. Especially in children, neuroinvasion of *Toxocara* larvae has been associated with mental confusion and cognitive impairment, as well as deficits in the development of speech and poor reading achievements, a higher distractibility, and lower intelligence in kindergarten children [17,18,37,38].

Similar to age as an influencing factor, the impact of sex is controversially discussed. The majority of studies have identified males as being at higher risk for *Toxocara* exposure [24,39,40], but females are at risk, too [41]. For males, the increased infection risk has been explained by a higher exposure to *Toxocara* eggs due to increased agricultural activities, and thus increased contact with *Toxocara* egg-contaminated soil and stray animals [42,43]. In the indigenous Wiwas, the seroprevalence did not differ statistically significant between sexes, as all inhabitants are presumed to be frequently exposed to *Toxocara* eggs.

Overall, the high seroprevalence of 79.3% in indigenous Wiwa indicates a high health risk of exposed people [19]. As infections might be accompanied by severe and detrimental symptoms, interventions and preventive measurements are necessary to eliminate the existing infection cycles. Although the installation of sanitary facilities remains difficult in these secluded villages, improved access to clean drinking water, and education in and implementation of adequate hygiene measures, such as washing of hands after soil contact or thorough washing of vegetables, are essential to prevent transmission [44,45]. Furthermore, the substantial reduction in infective *Toxocara* eggs in community environments would be an important measure. This comprises the avoidance of pet defecation in the villages and farmland and/or disposal of feces in these places [46,47]. However, avoiding defecation in crucial areas seems unrealistic, as animals are free-roaming and not housed and walked. Similarly, appropriate removal of feces, which is an effective measure to reduce environmental contamination, is not feasible to implement, as the contact with animal excrements is regarded as impure in the indigenous culture of the Wiwa. Frequent deworming of dogs and cats could be another approach to reduce environmental contamination, but this requires the provision of anthelmintics in sufficient quantities by governments or aid agencies.

## 5. Conclusions

The secluded life of the indigenous tribe of the Wiwas in north Colombia is characterized by, for example, a lack of access to clean drinking water and adequate hygiene standards. These well-known risk factors for zoonotic *Toxocara* infections are potentiated by nutrition habits and free-roaming dogs and cats in the Wiwa communities, resulting in a high seroprevalence of about 80% in the tested inhabitants. Deviating climatic conditions in the included villages are most likely responsible for the higher seroprevalences in communities above 1000 MASL with a subtropical or premontane humid forest compared to the lower altitudes with up to six months of dry season. Nevertheless, frequent *Toxocara* exposure is a common feature in all Wiwa communities, reflected by a cumulative effect with highest seroprevalences in adult inhabitants, and lacking sex differences. As toxocarosis can result in detrimental effects and long-term consequences, raising awareness thereof, and intervention measures, such as improvement in hygienic standards and reduction in soil contamination, are urgently needed in indigenous communities such as the Wiwa tribe.

## Figures and Tables

**Table 1:** *Toxocara* seroprevalences including 95% confidence intervals (CI) in the Wiwa community regarding different risk factors.

Group	Sample Size	Seropositive	Seroprevalence	95% CI
**Total**	483	383	79.3%	75.5–82.7%
**Villages**				
Tezhumake	173	122	70.5%	63.3–76.8%
Ashintukwa	106	79	74.5%	65.5–74.5%
Cherua	94	83	88.3%	80.3–93.3%
Siminke	110	99	90.0%	83.0–94.3%
**Age**				
children	75	50	66.7%	55.4–76.3%
adolescents	217	165	76.0%	77.9–87.5%
adults	191	168	88.0%	82.6–91.8%
**Sex**				
males	247	187	75.7%	70.0–80.6%
females	232	193	83.2%	77.9–87.5%
**MASL**				
below 1000 MASL	279	201	72.0%	66.5–77.0%
above 1000 MASL	204	182	89.2%	84.2–92.8%

Abbreviation: MASL, meters above sea level.

**Table 2:** Results from the general linear model (GLM) testing the effect of different variables on the *Toxocara* seroprevalence in the North Colombian indigenous tribe of the Wiwa (*n* = 483). The final model was significantly different from a null model containing only an intercept term (likelihood ratio test, df = 6, χ^2^ = 58.643, *p* < 0.001). Significant *p*-values are printed in bold.

Variable	Estimate	Standard Error	z-Value	*p*-Value
Intercept	2.587	0.3921	6.598	**<0.001**
**Village**				
Ashintukwa vs. Cherua	0.903	0.441	2.049	0.165
Ashintukwa vs. Siminke	1.398	0.457	3.061	**0.012**
Ashintukwa vs. Tezhumake	−0.500	0.317	−1.576	0.384
Cherua vs. Siminke	0.495	0.523	0.946	0.775
Cherua vs. Tezhumake	−1.403	0.410	−3.423	**0.003**
Siminke vs. Tezhumake	−1.898	0.433	−4.388	**<0.001**
**Age**				
children vs. adolescents	−0.852	0.326	−2.617	**0.024**
children vs. adults	−2.099	0.400	−5.248	**<0.001**
adolescents vs. adults	−1.246	0.333	−3.748	**<0.001**
**Sex**				
male vs. female	−0.325	0.261	−1.246	0.213
**MASL**				
below vs. above 1000 MASL	−1.458	0.306	−4.773	**<0.001**

Multiple comparisons between the levels of the factors “village”, “age”, and “sex” were calculated using Tukey contrasts with single-step *p*-value adjustment. The comparison of MASL was calculated separately (null model containing only an intercept term with likelihood ratio test, df = 4, χ^2^ = 55.219, *p* < 0.001). Abbreviation: MASL, meters above sea level.

## Data Availability

Data supporting reported results are contained within the article.
